# Improving Person-Centered Access to Dental Care: The Walk-In Dental Encounters in Non-Emergency Situations (WIDENESS)

**DOI:** 10.3390/dj7040116

**Published:** 2019-12-13

**Authors:** Noémie Gulion, Jean-Noel Vergnes

**Affiliations:** 1 Faculty of Dentistry, Dental Public Health Department, University Hospital of Toulouse, 3, chemin des maraîchers, 31400 Toulouse, France; noemiieg3@gmail.com; 2 Faculty of Dentistry, McGill University, 2001 McGill College Ave, Montreal, QC H3A 1G1, Canada

**Keywords:** person-centered care, dentistry, oral health needs, access to oral care, walk-in consultations, prevention

## Abstract

Background: We hypothesized that access to dental care could be improved by the conceptualization of a new type of consultation: The walk-in dental encounter for non-emergency situations (WIDENESS). The aim of this study was to assess patient perspectives regarding walk-in dental consultations, with a particular focus on non-emergency situations. Methods: We followed a qualitative research approach using a semi-structured interview guide in a sample of random participants recruited from the dental department of the Toulouse University Hospital, France. We performed a thematic analysis of the interview transcripts. Data saturation was obtained after interviewing 11 participants. Results: When asked about walk-in dental consultations, three main topics emerged: (1) Walk-in dental consultation in general is important for emergency situations, but WIDENESS did not correspond to any specific long-standing need from participants; (2) WIDENESS could be a way to improve access to oral care (facilitating access to care relative to time constraints, reduction of dentist-related anxiety, better overall follow-up for the care pathway, and the complementary nature of consultations with and without appointments); and (3) WIDENESS has some potential drawbacks—apprehension about long waiting times was mentioned by several participants. Conclusions: Participants found the idea of WIDENESS promising, despite spontaneously mentioned reservations, which constitute major challenges to its implementation.

## 1. Introduction

Access to dental care is a major determinant of oral health [1]. At the societal level, the economic environment plays a crucial role in access to dental care [2]. At the individual level, it is admitted that access to dental care depends on both the availability of oral health services and the patient’s willingness to seek oral health care [3]. As a complex, multilevel problem, there is no universal and single-factor solution to improve access to dental care [3].

Among various improvement avenues, the organization of the dental work force should evolve with changing perceptions of individuals regarding health in modern societies. In dentistry—as in medicine—there is a trend toward person-centered care (PCC) [4], reflecting the wishes of individuals to have more power and control in their health decisions [5]. Patient-centeredness (a close relative concept to person-centeredness) is now strongly encouraged by various institutions, such as the Commission on Dental Accreditation in the United States [6], the Association of Canadian Faculties of Dentistry [7], or the Association for Dental Education in Europe [8]. There are now concrete implementations of person-centeredness in dental curricula [9] and in day-to-day clinical practice [10]. However, the question of how access to dental services could also embrace a person-centered direction remains to be conceptualized, implemented, and evaluated.

Nowadays, the main locations for dental practice are usually a private dental office/clinic or public hospital dental departments [11]. In these places, dental practice is traditionally organized around two types of consultations: Regular appointments (first or follow-up visit) and emergency consultations (walk-in hours for offices, or walk-in emergency units in hospitals). In this article, we hypothesize that access to dental care could be improved by the conceptualization of a third type of consultation: The walk-in dental encounter for non-emergency situations. To initiate the debate on this innovative topic, the aim of this study was to assess patient perspectives regarding walk-in dental consultations, with a particular focus on non-emergency situations.

## 2. Materials and Methods

### 2.1. Terminology

Here we define some key notions:

(a) Scheduled dental encounter: An encounter between a patient and an oral health professional that occurs within time slots for scheduled one-on-one visits (i.e., “Patient X has an appointment with Dr Y at hh:mm, mm/dd/yyyy”).
(b) Walk-in dental encounter: An encounter between a patient and an oral health professional that occurs within specific time slots for unscheduled visits (i.e., Patient X goes to Dr Y’s office, who “consults without appointment every Thursday between hh:mm and hh:mm”).
(c) Dental emergency: Any situation usually perceived by both patients and healthcare professionals as of sufficient concern to require a quick intervention (i.e., within 24 h) [12,13]. For example, dental emergencies such as acute dental pain, orofacial trauma, acute infections, active bleeding, or loss of prosthetic anterior teeth [12].
(d) Non-emergency dental situations: All other situations—excluding (c)—when patients perceive a need for any dental health service, ranging from a relative dental emergency (e.g., problem with prosthetic posterior teeth or chronic pain [12]) to a dental check-up.

Considering (b) and (d) together, we refer in the next paragraphs to the acronym “WIDENESS” to designate the “Walk-In Dental Encounters in Non-Emergency SituationS”.

### 2.2. Preliminary Quantitative Survey

In order to probe the relevance of our research question in the general population, and eventually to enhance the interpretation and meaningfulness of our qualitative analysis [14], we first conducted a preliminary quantitative survey. This survey consisted of three basic questions: (1) Have you ever given up on receiving dental care because you were not able to get an appointment? (2) Would you be more likely to solicit dental check-ups if dental offices offered walk-in consultations? (3) Would walk-in consultations improve your access to dental care? The survey was conducted using an online questionnaire, in a convenience sample of French adults. Given a population of French adults of *N* = 50 million, and using an 80% confidence interval and a 5% sampling error, a minimum sample of 164 participants was required. A total of 229 participants answered the questions (among whom 64% were women, 49% were less than 30 years old, and 76% were from the Occitanie region, in the southwest of France). Despite the significant possibility of both a social desirability bias and a selection bias due to the use of an Internet survey, the high proportion of “yes” responses for the three questions (29.3%, 61.6%, and 68.6%, respectively) indicated it was warranted that patient perspectives on walk-in dental consultations be explored further. We thus designed a qualitative research study, as detailed below.

### 2.3. Study Design and Sampling Strategy

To assess patient perspectives on walk-in dental consultations, we used qualitative methodology, as this allows exploration of phenomena about which little is known [14]. We followed the Standards for Reporting Qualitative Research (SRQR) guidelines for qualitative studies [15]. In order to let patients express themselves openly on the topic of walk-in dental consultations in general, interviews were initiated on this topic. If not brought up spontaneously by the participant, WIDENESS was then suggested by the interviewer, following a semi-structured interview guide. We conducted individual interviews with a sample of patients recruited from the dental department of the Toulouse University Hospital (CHU de Toulouse, services d’Odontologie de Rangueil et de l’Hôtel-Dieu). Participants were randomly chosen and were included on a voluntary basis at the end of their dental consultation (that could be initial consultations or follow-up visits).

### 2.4. Ethical Considerations

Participants were informed about the purpose of the research and about the nature of the collected and analyzed data (audio recordings with transcription of interviews). They were also informed about confidentiality and had the right to withdraw from the study at any time. Information was provided orally using a non-technical language. Before the interview, participants were asked to sign a consent form. The transcripts were edited to maintain the anonymity of the participants and of their entourage. Confidentiality of data (audio recordings and transcripts) was assured. This research was approved by the Institutional Review Board of the Toulouse Dental Department (10_20_2017_TOU3 3061) on 20 October 2017.

### 2.5. Data Collection

One interviewer (N.G.) collected data between March and May 2017. The interviews were carried out in a quiet room at the dental unit. The interviewer (a full-time final year dental student) was trained in qualitative methods and ensured the discussion did not stray off topic and led discussions in a conversational tone. All interviews were recorded digitally, and later transcribed fully by the interviewer. Data were collected until data saturation was reached, i.e., when no new information was obtained [14]. Indeed, as a qualitative study design, we aimed to reach an informationally representative sample (i.e., not a statistically representative sample).

### 2.6. Data Management and Analysis

We performed a thematic analysis, as described by Braun and Clarke [16]. The process involved separating the transcripts into meaningful segments and assigning codes to the segments. Coding of the interview transcripts was carried out using Microsoft Word’s comment feature. Codes and their corresponding passages were then regrouped into broad themes and displayed in an analytic matrix [17]. Once the themes were identified, we described them in a text and illustrated them with transcript excerpts [17].

### 2.7. Description of the Sample

Data saturation was obtained after interviewing 11 participants (15 patients were asked to participate to reach the sample size of 11). The mean age of the sample was 61.3 years old and there were 8 women and 3 men. Table 1 summarizes the participants’ characteristics.

## 3. Results

When asked about walk-in dental consultations, three main topics emerged: (1) Walk-in dental consultation in general is important for emergency situations, (2) WIDENESS could be a way to improve access to oral care, and (3) WIDENESS has some potential drawbacks.

### 3.1. Walk-in Dental Consultation is Important for Emergency Situations

Walk-in dental consultations were associated solely with emergency care, and considered as being tremendously important by all participants. For some participants, the perception of a dental emergency is the main reason to consult a dentist. WIDENESS did not correspond to any specific long-standing need from all participants. Thus, participants did not spontaneously bring up WIDENESS as a way to improve their access to dental care (Table 2).

### 3.2. WIDENESS Could be a Way to Improve Access to Oral Care in Non-Emergency Situations

After the concept of WIDENESS was suggested to participants, some of them found the idea interesting to improve access to oral care. They put forward different arguments, such as facilitating access to care relative to time constraints, reduction of dentist-related anxiety, better overall follow-up for the care pathway, and the complementary nature of consultations with and without appointments (Table 3).

### 3.3. WIDENESS Has Potential Drawbacks

Although some participants were very enthusiastic toward WIDENESS, others pointed out some potential drawbacks associated with this type of consultation. Apprehension about long waiting times is mentioned by several participants, along with the fear of unsuccessful appointments and even general skepticism about the concept (Table 4).

## 4. Discussion

### 4.1. Summary and Key Findings

Although walk-in dental consultations were spontaneously and exclusively associated with dental emergencies by the participants, it seems that the concept of walk-in dental encounters for non-emergency situations—once presented to them—opens interesting opportunities for improving access to oral care. According to patients’ perspectives, WIDENESS could be a way to “expand” access to dental care, even if some important issues remain to be solved.

### 4.2. Strengths and Limitations

The main strength of the study lies in its exploration of a new type of dental encounter, which has never been described. Another strength is that the flexibility of qualitative interviews allows to obtain balanced information on the topic. Our study also has several limitations that should be mentioned. The main limitation is the relative superficiality of the themes collected. In qualitative research, there are various levels of analysis in terms of depth, ranging from superficial description to theoretical interpretation [18]. As our research question was related to a very specific issue—and could not be the subject of an experiential report by participants because of its novelty—more in-depth exploration was neither desirable nor possible. This may explain why interviews were relatively short, and data saturation was obtained sooner than expected. Another limitation was that practitioners’ perspectives on WIDENESS were not explored in this study. Although this was intentionally foreseen in the design of the study, their views should be collected before implementing WIDENESS. Indeed, certain crucial aspects such as the economic viability of such consultations should be discussed, conceptualized, and analyzed. A good way to initiate the practical implementation of WIDENESS could be to use practice-based research networks that are currently flourishing in dentistry. Finally, the generalizability of our findings is limited because the sample was drawn from a university hospital that holds a large proportion of retirees; to what extent our findings might relate to younger populations is therefore unclear. However, the responses obtained in our preliminary survey indicate that younger people might also find some interests in WIDENESS (and maybe even more so because of working time constraints). The fact remains that these results may not be generalizable to populations from outside southwest France.

### 4.3. Interpretation and Implications

Walk-in clinics are not a new concept in medical or dental care. The novelty of WIDENESS resides in dissociating the walk-in concept from emergency care in dentistry, which has never been described in the literature. It is likely that WIDENESS-like consultations have already been implemented in some dental settings across the world, as a local response to local demands; but to the best of our knowledge, it has not been conceptualized yet in the dental literature. In our opinion, this lack of description in the literature limits the emergence of this type of consultation.

Some important features regarding the results of this study need to be discussed relative to current knowledge and traditional dentistry practice.

The fact that participants did not spontaneously talk about the possibility of WIDENESS represents a major barrier to its implementation. Scheduling habits in dental modern practice may act as a strong shaper of participants’ thoughts about access to dental care (P2: “most doctors simply work by appointment, that’s all”). The deployment of WIDENESS would thus require significantly raising patient awareness. Due to the novelty of this type of consultation in dentistry, websites for dental care offices or clinics should thus clearly present the framework of the WIDENESS activity: What it is and what it is not. The internet provides an opportunity for dentists to showcase their practice philosophy, quality of care, office setting, and staff [19], and also, to explain how appointment time slots are allocated. Distinction with the emergency activity should be presented as clearly as possible (for example, by giving examples of what a dental emergency is, in the context of the particular dental setting). This clear distinction would be particularly important, since even the provision of dental emergency care is perceived as insufficient in some studies [20,21]. Patient information should deal with the fact that WIDENESS only involves some predefined types of treatments (whose list may be determined based on local demands, communities, and practices), such as dental check-ups with no need for follow-up, requests for specific advice about oral health, or routine periodontal or prosthodontics maintenance.

Our results also show that another major barrier to implementation of WIDENESS is the fear of crowded waiting rooms—a legitimate fear that is very likely to be shared by oral health professionals as well. It is true that crowded waiting rooms are stressful for both patients and medical teams [22]; however, the traditional scheduled dental encounter is not a perfect solution. There are at least three problems with the planned scheduling system [23]: (1) Appointments often take longer than expected, (2) patients often show up late to their appointments, and (3) delays are cumulative throughout the day—one late appointment could make every other appointment late. We thus argue that synergistically diversifying the scheduling organization for non-emergency situations would allow patients to benefit from the strengths of both systems, while minimizing their respective limitations. Again, modern technologies could provide useful tools to help practitioners to provide better PCC [24]. In the context of WIDENESS, easy-to-implement applications could provide real-time estimates of the occupancy rate of waiting rooms during WIDENESS time slots. Finally, it should be clearly acknowledged as an implied agreement that recourse to WIDENESS time slots exposes one to an unavoidable probability of waiting (which is less of a concern than in emergency situations).

Several advantages may be associated with the development and dissemination of WIDENESS in the dental setting.

First, WIDENESS could indirectly enhance access to emergency care and to the scheduled dental visits as well. Indeed it has been shown that many patients use emergency dental services as a way to avoid the long waiting periods associated with waiting lists for general dental care [13]. Without intermediate solutions such as WIDENESS, both traditional scheduled dental care and dental emergency services are working in suboptimal ways. For example, it has been shown that medical walk-in clinics may have the potential to reduce non-urgent emergency presentations [25].

Second, WIDENESS could represent a way to handle some complex real-life situations that happen in day-to-day dental practice. For example, it is likely that WIDENESS would allow practitioners to better handle the uncertainty linked with situations they face daily: Optional follow-up appointments following a procedure with low risk of complications (surgical dentistry, simple tooth extraction, etc.), proposal for another appointment strategy for patients that are frequently late, or handling of patients’ worries (semi-urgent cases) within a reasonable time frame (2–7 days) [12], including communication on the telephone [26]. WIDENESS timeslots could also allow dentists to analyze observations from teledentistry examinations [27]. More generally, such organization would allow dentists to “manage patient singularity on a large scale” [28], helping them to provide “socially competent” services [29,30].

Third, this kind of organization fits well with societal changes related to healthcare, and with the patients’ desire for greater autonomy. WIDENESS may provide new opportunities for the dental community to offer to patients more flexible access to dental services—to a certain extent, depending of structural constraints. From a PCC perspective, such a service could improve access to care by facilitating the dental care pathway described by Grembowski [31]. In particular, walk-in consultations could encourage new asymptomatic patients to make spontaneous visits to a dental professional (thus promoting disease-prevention behaviors), and minimize dental care renunciation because of professional constraints of the patient—a significant cause of care renunciation that has been documented in France [32].

Fourth, always following the PCC principles, and among them the dentist-as-person concept [4,10], we also hypothesize that adding some WIDENESS timeslots into professional agendas could result in better working conditions for dentists. It has been shown that dentists have to deal with many significant stressors in their professional lives [33]. Among them, dentists often indicate that “running behind schedule” is one of the most stressful factors associated with daily dental practice [34,35]. It is also likely that other barriers to person-centered care would prevent dentists to provide WIDENESS timeslots [36].

### 4.4. Controversies Raised by the Study and Future Research Directions

Better access to oral health care implies some structural changes in the supply of care. Participants in our study found the idea of WIDENESS promising, despite spontaneously mentioned reservations, which constitute major challenges to its implementation. In an optimal organization, it is likely that the contribution of WIDENESS in dentistry should be limited to a small proportion of all consultations, whether in relation to the types of procedures or the volume of time devoted to them. Moreover, the structuring of WIDENESS should not lead to a deterioration of the existing offer, whether for emergency slots or those of scheduled visits. To our knowledge, our study is the first to investigate patients’ perspectives regarding walk-in dental consultations for non-emergency situations, which is an important first step toward further conceptualization and implementation on the topic. Future research is needed in order to assess different modalities of implementation.

## Figures and Tables

**Table 1:** Description of participants’ characteristics.

# Id	Gender	Age
***P1***	Male	67 years old
***P2***	Male	Not reported
***P3***	Female	60 years old
***P4***	Female	26 years old
***P5***	Female	71 years old
***P6***	Female	61 years old
***P7***	Male	71 years old
***P8***	Female	72 years old
***P9***	Female	77 years old
***P10***	Female	37 years old
***P11***	Female	71 years old

**Table 2:** Participants’ quotations, illustrating their spontaneous point of view regarding walk-in dental consultations in general.

Themes	Quotations
Walk-in consultations as the only way of consulting	*P1: First visit because of an emergency, second visit because I had to go there, a third one to settle what I owed, and then he never saw me again. […]. Usually when we go to see them, that’s because we’re not doing very well.* *P4: I do everything at the last minute, all in a hurry.*
Walk-in dental consultations are integrated into emergency consultations	*P4: what’s super cool is that they performed the procedure now right away because […], you know, I was really in pain.*
What is not urgent can wait for an appointment (no need for walk-in consultation for routine dental treatments)	*P9: The scale removal for example … no … I think we can wait a little bit [for an appointment]. We can wait because we can see it coming.*

**Table 3:** Participants’ quotations, illustrating their point of view regarding positive aspects of walk-in dental encounters in non-emergency situations.

Themes	Quotations
Facilitating access to care relative to time constraints	*P4: In fact, since I don’t have a lot of free time because of my job—I’m free from 1:00 p.m. to 5:00 p.m.—I tell myself I have my afternoon there […], I’ll wait in the waiting room. I know that I’ll be received […] and that’s it …* *P10: It means that when you have a need, you can find a solution […] in a quite reasonable time.*
Reduction of dentist-related apprehension	*Interviewer: If a dentist offered walk-in visits in non-emergency situations, would it help patients come to the dentist more often?* *P6: I think so.* *Interviewer: Why?* *P6: I don’t know, to [reduce] the fear of the appointment, the fear of the dentist …*
Better overall follow-up in the pathway of care	*P10: I think that if there were more ease, accessibility [thanks to] walk-in dentists, […] there would be less problems with follow-up care afterwards.*
Complementary nature of consultations with and without appointment	*P5: That is to say, it’s nice on the one hand to go to consultations without an appointment, and it’s nice to have appointments too.* *P2: The fact that there are also some kinds of consultations without appointment, yeah it might be much better.*

**Table 4:** Participants’ quotations, illustrating potential drawbacks of walk-in dental encounters in non-emergency situations.

Themes	Quotations
Fear about long waiting times	*P1: I don’t like it too much because this is the day when everyone arrives and then you realize that you are the 78th to be taken … or it is necessary to arrive 4 h before the opening of the practice to be sure in the first to be received. So I definitely prefer the appointment consultations.* *P7: if, on the other hand, I arrive in a waiting room full of people like me hoping to be received quickly, the goal would not be reached.*
Fear of an unsuccessful appointment	*P11: Well, provided that we don’t go [to the dentist] for nothing.* *P7: I [prefer] appointments, hoping that people will be on time rather than going there and wait in vain.*
Scepticism about the concept (no room for other types of consultations)	*P9: In principle, this is not how it works.* *P5: [People] have a toothache: they come to be cared for … and then we never see them again. If you go to an appointment, you know there will be a follow-up. I think it’s not the same thing.*
